# Anthelmintic mebendazole enhances cisplatin's effect on suppressing cell proliferation and promotes differentiation of head and neck squamous cell carcinoma (HNSCC)

**DOI:** 10.18632/oncotarget.14673

**Published:** 2017-01-16

**Authors:** Fugui Zhang, Yong Li, Hongmei Zhang, Enyi Huang, Lina Gao, Wenping Luo, Qiang Wei, Jiaming Fan, Dongzhe Song, Junyi Liao, Yulong Zou, Feng Liu, Jianxiang Liu, Jiayi Huang, Dan Guo, Chao Ma, Xue Hu, Li Li, Xiangyang Qu, Liqun Chen, Xinyi Yu, Zhicai Zhang, Tingting Wu, Hue H. Luu, Rex C. Haydon, Jinlin Song, Tong-Chuan He, Ping Ji

**Affiliations:** ^1^ Chongqing Key Laboratory for Oral Diseases and Biomedical Sciences, and the Affiliated Hospital of Stomatology of Chongqing Medical University, Chongqing, China; ^2^ Molecular Oncology Laboratory, Department of Orthopaedic Surgery and Rehabilitation Medicine, The University of Chicago Medical Center, Chicago, IL, USA; ^3^ Ministry of Education Key Laboratory of Diagnostic Medicine, and the Affiliated Hospitals of Chongqing Medical University, Chongqing, China; ^4^ Department of Conservative Dentistry and Endodontics, West China Hospital and West China School of Stomatology, Sichuan University, Chengdu, China; ^5^ Department of Orthopaedic Surgery, Union Hospital of Tongji Medical College, Huazhong University of Science and Technology, Wuhan, China; ^6^ Department of General Surgery, The Affiliated Zhongnan Hospital of Wuhan University, Wuhan, China; ^7^ Department of Biomedical Engineering, School of Bioengineering, Chongqing University, Chongqing, China; ^8^ Department of Neurosurgery, The Affiliated Zhongnan Hospital of Wuhan University, Wuhan, China

**Keywords:** head and neck squamous cell carcinoma, mebendazole, drug repurposing, keratinization, differentiation therapy

## Abstract

Head and neck squamous cell carcinoma (HNSCC) is one of the most common and aggressive types of human cancers worldwide. Nearly a half of HNSCC patients experience recurrence within five years of treatment and develop resistance to chemotherapy. Thus, there is an urgent clinical need to develop safe and novel anticancer therapies for HNSCC. Here, we investigate the possibility of repurposing the anthelmintic drug mebendazole (MBZ) as an anti-HNSCC agent. Using the two commonly-used human HNSCC lines CAL27 and SCC15, we demonstrate MBZ exerts more potent anti-proliferation activity than cisplatin in human HNSCC cells. MBZ effectively inhibits cell proliferation, cell cycle progression and cell migration, and induces apoptosis of HNSCC cells. Mechanistically, MBZ can modulate the cancer-associated pathways including ELK1/SRF, AP1, STAT1/2, MYC/MAX, although the regulatory outcomes are context-dependent. MBZ also synergizes with cisplatin in suppressing cell proliferation and inducing apoptosis of human HNSCC cells. Furthermore, MBZ is shown to promote the terminal differentiation of CAL27 cells and keratinization of CAL27-derived xenograft tumors. Our results are the first to demonstrate that MBZ may exert its anticancer activity by inhibiting proliferation while promoting differentiation of certain HNSCC cancer cells. It's conceivable the anthelmintic drug MBZ can be repurposed as a safe and effective agent used in combination with other frontline chemotherapy drugs such as cisplatin in HNSCC treatment.

## INTRODUCTION

Head and neck squamous cell carcinoma (HNSCC) is the sixth most common cancer by incidence worldwide with an annual incidence of approximately 600,000 cases [1–3]. The overall survivals at two and five years are 70% and 55%, but the recurrence-free survivals at two and five years are only 60% and 52%, respectively [3, 4]. Thus, it is urgent to improve the five-year survival rate of HNSCC patients. Clinically, cisplatin is used as one of the first-tier chemotherapeutic drugs against human HNSCC although its therapeutic effect has been less than satisfactory [5]. HNSCC patients receiving chemotherapy still proceed to the advanced stage, cancer local recurrence, or metastasizing to regional lymph nodes and/or distant organs largely due to the development of resistance to cisplatin and/or other agents [6, 7]. Therefore, there is an urgent clinical need to develop novel and effective anticancer agents for efficacious HNSCC treatment.

Repurposing previously approved drugs for cancer treatment represents an attractive, safe and economical strategy for cancer drug discovery. Mebendazole (MBZ) has been one of the most successful antiparasitic drugs and is used extensively in anthelmintic therapy as MBZ has been approved by the US FDA to treat parasitic infections and has a long track-record of safe profiles for human use and in various animal models [8]. Several studies indicate that MBZ and/or its derivative flubendazole exerted anticancer activities in several types of human cancers [8–20]. However, it is not clear if MBZ exhibits any cytotoxicity in HNSCC cancer cells.

Here, we investigate whether MBZ exerts any anticancer activity and synergizes with the chemotherapy agent cisplatin in HNSCC cancer cells. Using the two commonly-used HNSCC lines CAL27 and SCC15 [21–23], we demonstrate that MBZ markedly inhibits cell proliferation and induces apoptosis in HNSCC cells. MBZ is shown to exhibit synergistic anticancer activity with cisplatin. MBZ promotes the terminal differentiation of CAL27 cells and keratinization of CAL27-derived xenograft tumors.

## RESULTS

Mebendazole (MBZ) exerts more potent anti-proliferation activity than cisplatin (CIS) in human head and neck squamous cell carcinoma (HNSCC) cells

**Figure 1:**
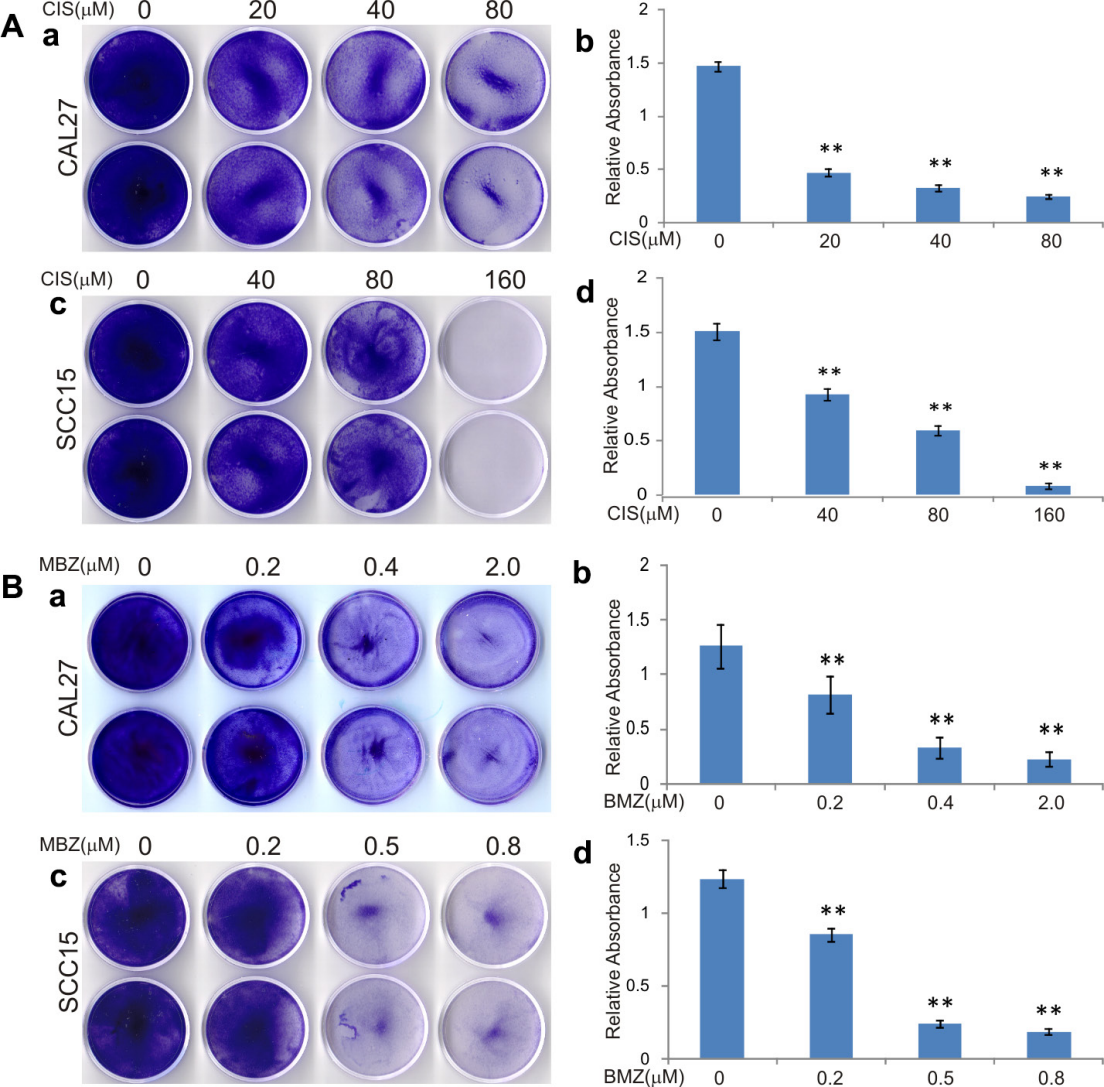
Mebendazole (MBZ) exerts more potent anti-proliferation activity than cisplatin (CIS) in human head and neck squamous cell carcinoma (HNSCC) cells Subconfluent HNSCC cell lines CAL15 and SCC15 were treated with CIS (**A**) or MBZ (**B**). At 3 days after treatment, the cells were fixed and stained with crystal violet (a and c), followed by a quantitative analysis of absorbance of the stained viable cells dissolved in acetic acid (b and d). Each assay condition was done in triplicate. Representative results are shown. **p < 0.001.

We first compared the anti-proliferative effect of mebendazole (MBZ) with that of the clinically used chemotherapeutic agent cisplatin (CIS) in two commonly-used human HNSCC lines CAL27 and SCC15. When these lines were treated with cisplatin, a dose-dependent inhibition of cell proliferation was observed (Figure 1A) and a significant inhibitory effect was seen at 20 μM and 40 μM for CAL27 and SCC15 cells, respectively, although CAL27 cells were more sensitive to cisplatin than SCC15 cells (Figure 1A ab vs. cd). When these cells were treated with MBZ, CAL27 cell proliferation was inhibited at 0.2 μM MBZ, or more pronouncedly at 0.4 μM (Figure 1B-ab), while SCC15 cells were markedly inhibited at 0.5 μM MBZ (Figure 1B-cd). These *in vitro* results demonstrate that MBZ exhibits more potent anti-proliferation activity in HNSCC cells than that of cisplatin's. Furthermore, SCC15 cells were shown relatively insensitive to cisplatin, but can be effectively inhibited by MBZ at low concentrations, suggesting that a combination of MBZ and cisplatin may act more effectively on inhibiting HNSCC cell proliferation.

### MBZ effectively inhibits cell proliferation and cell cycle progression and induces apoptosis of human HNSCC cells

**Figure 2:**
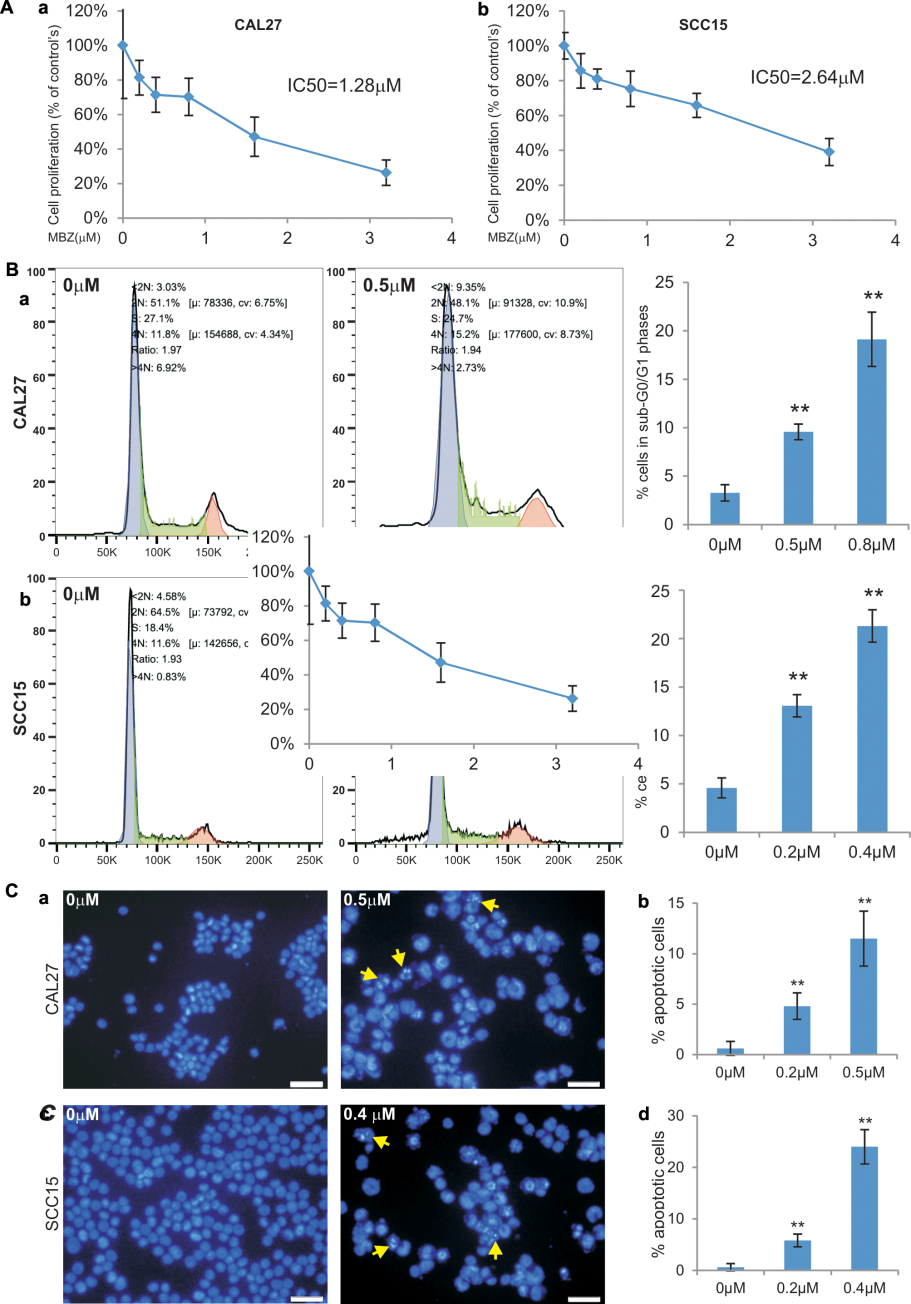
MBZ effectively inhibits cell proliferation and cell cycle progression and induces apoptosis of human HNSCC cells (**A**) Subconfluent HNSCC cell lines CAL15 (a) and SCC15 (b) were treated with MBZ at the indicated concentrations for 24 h and incubated with premixed WST-1 reagent for 2 h before measuring absorbance. IC50 was calculated for each line. Each assay condition was done in triplicate. (**B**) Subconfluent CAL15 (a) and SCC15 (b) were treated with MBZ at the indicated concentrations for 24 h and collected for cell cycle analysis. The % cells accumulated in sub-G0/G1 phases were calculated. **p < 0.001. (**C**) Subconfluent CAL15 (a and b) and SCC15 (c and d) were treated with the indicated concentrations of MBZ for 24 h and fixed and stained with Hoechst 33258. The % apoptotic cells (indicated by arrows) were calculated by counting at least 10 high power fields (B and D).

We further evaluated anti-proliferative effect of MBZ using the more sensitive and quantitative WST-1 proliferation assay. When subconfluent CAL27 and SCC15 cells were treated different concentrations of MBZ, a significant inhibition of cell proliferation was observed at concentrations as low as 0.4 μM MBZ in CAL27 (*p* < 0.01) and 0.2 μ M MBZ in SCC15 (*p* < 0.05) (Figure 2A-ab). The calculated IC50 values are 1.28 μM and 2.64 μM for CAL27 and SCC15 cells, respectively (Figure 2A). Thus, the WST-1 assay results are largely consistent with that were obtained from the crystal violet staining assay shown in Figure 1.

We also examined the effect of MBZ on cell cycle progression. When CAL27 cells were treated 0.5 μM or 0.8 μM MBZ, we found the percentage of cells accumulated in sub-G0/G1 phases increased significantly (*p* < 0.001) (Figure 2B-a). Similarly, MBZ treatment of SCC15 cells at rather low concentrations (0.2 μM or 0.4 μM) even led to more significant accumulations of sub-G0/G1 cells than that for CAL27 cells’ (Figure 2B-b). These results suggest that MBZ-inhibited HNSCC cell proliferation may at least in part cause by suppressing cell cycle progression.

To understand the possible mechanism underlying MBZ-induced inhibition of cell proliferation, we also investigated the effect of MBZ on inducing apoptosis in HNSCC cells. Treatment of CAL27 cells with 0.2 μM or 0.5 μM MBZ induced significant apoptosis (*p* < 0.001) (Figure 2C-ab). SCC15 cells were even more sensitive and MBZ induced significant apoptosis at low concentrations (0.2 μM or 0.4 μM) (*p* < 0.001) (Figure 2C-cd). Thus, these results demonstrate that MBZ is a potent apoptosis inducer for HNSCC cells.

### MBZ inhibits cell migration of human HNSCC cells

**Figure 3:**
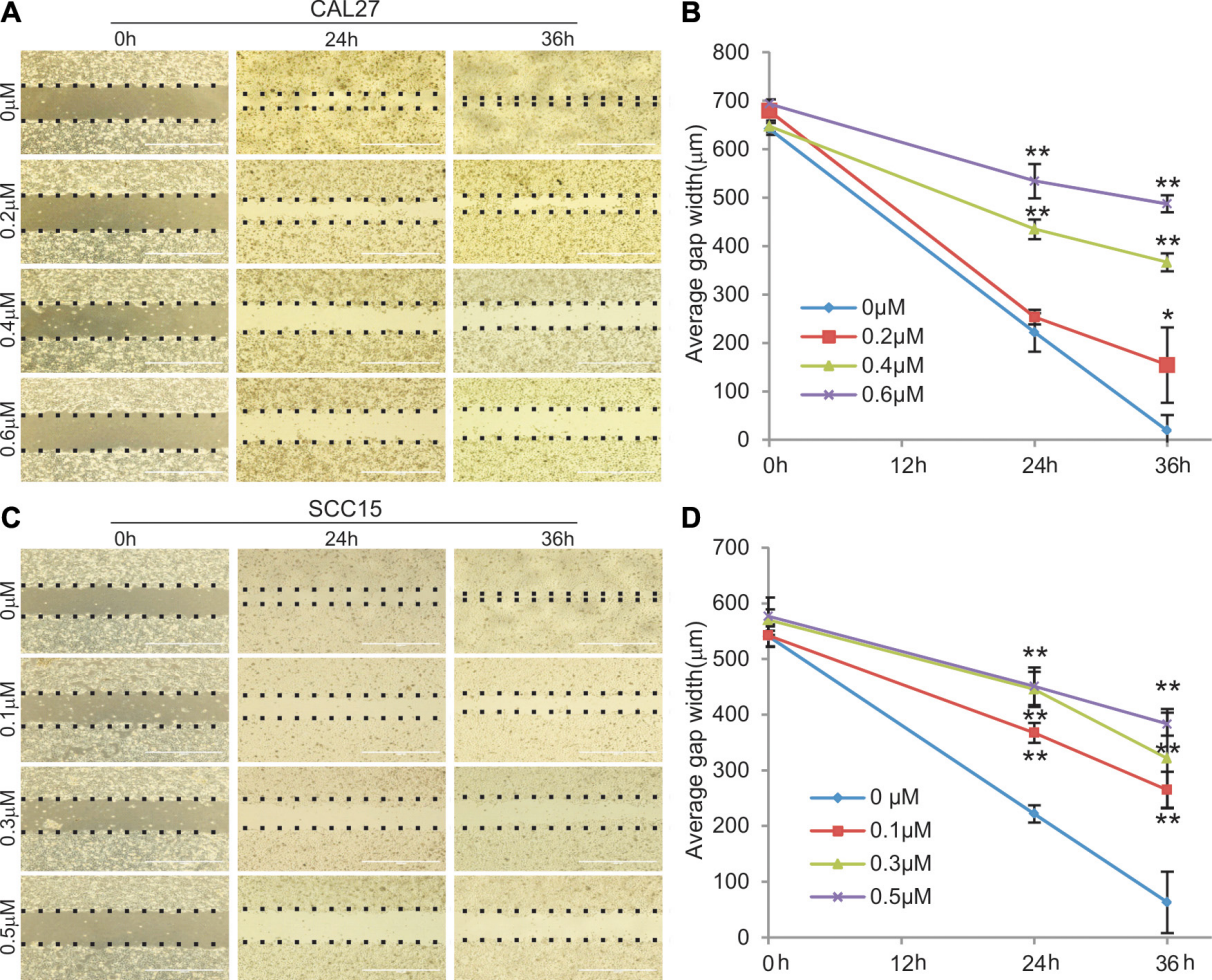
MBZ inhibits cell migration of human HNSCC cells Freshly confluent CAL15 (**A** and **B**) and SCC15 (**C** and **D**) were wounded with pipet tips and treated with MBZ at the indicated concentrations of MBZ and the gaps (dotted lines) were measured at the indicated time points. *p < 0.05; **p < 0.001.

We analyzed whether MBZ exerts any effect on cell migration and wound healing in HNSCC cells. When freshly confluent CAL27 monolayer cells were wounded and treated with 0, 0.2 μM, 0.4 μM, or 0.6 μM MBZ, we found the wound closure ratios were inversely correlated with MBZ concentrations; and the wound gaps failed to close in the presence of 0.4 μM and 0.6 μM MBZ even at 36 h after wounding while the control group healed completely (Figure 3-ab). Similar results were obtained in MBZ-treated SCC15 monolayer cells, except that the wound gaps failed to close even at lower concentrations of MBZ (0.3 μM and 0.5 μM) (Figure 3-cd). These results indicate that MBZ can inhibit cell wound healing and migration of HNSCC cells in a dose-dependent fashion.

### MBZ affects multiple cancer-related signaling pathways differently in CAL27 and SCC15 lines

**Figure 4:**
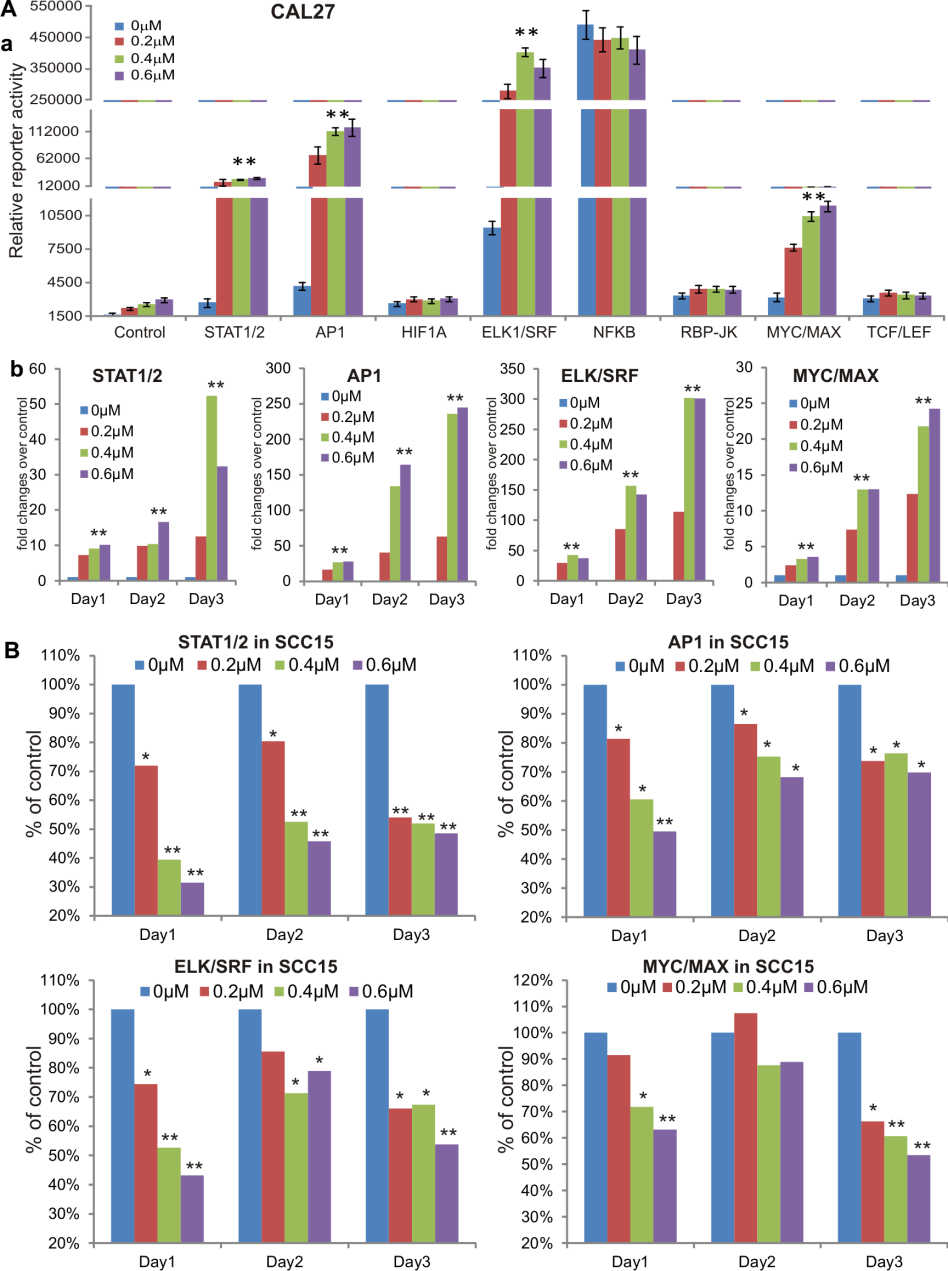
MBZ affects multiple cancer-related signaling pathways differently in CAL27 and SCC15 lines (**A**) MBZ activates multiple cancer-related signaling pathways in CAL27 cells. Subconfluent CAL27 cells were transfected with the indicated eight pathway reporters and treated with different concentrations of MBZ for 24 h. The Gaussia luciferase reporter activities were assessed (a). The four activated pathway reporters were further analyzed at 48 h and 72 h (b). (**B**) In SCC15 cells MBZ inhibits the four cancer-related signaling pathways that are activated in CAL27 cells. Subconfluent SCC15 cells were transfected with the indicated four pathway reporters and treated with different concentrations of MBZ. The Gaussia luciferase activities were assessed at 24 h, 48 h and 72 h. *p < 0.05, **p < 0.001.

To explore the possible mechanism underlying MBZ's antiproliferative activity, we tested the effect of MBZ on eight cancer-related pathway reporters in CAL27 and SCC15 cells. These cancer-associated pathways were previously characterized [24–26]. When Gaussia luciferase reporters for the eight pathways were transfected into CAL 27 cells and treated with 0, 0.2, 0.4 or 0.6 μM MBZ for 24 h, Gaussia luciferase activities of ELK1/SRF, AP1, STAT1/2 and MYC/MAX reporters were increased in a dose-dependent manner, while HIF1A, NFKB, RBP-JK and TCF/LEF reporter activities were not significantly affected (Figure 4A-a). We further extended the reporter assay time points and found that ELK1/SRF, AP1, STAT1/2 and MYC/MAX reporter activities were significantly increased in CAL 27 cells in MBZ-treated groups (0.2, 0.4 or 0.6 μM) in a dose-dependent manner, compared to that of 0 μM MBZ control group's (Figure 4A-b). Interestingly, when the same set of four pathway reporters was assessed in SCC15 cells, we found MBZ effectively inhibited these reporter activities in a dose-dependent fashion at most of the tested time points (Figure 4B). These results suggest that MBZ may exert its anticancer activity in a context-dependent manner although the precise mechanism through which MBZ suppresses cell proliferation of HNSCC cells remains to be thoroughly investigated.

### MBZ synergizes with cisplatin in suppressing cell proliferation and inducing apoptosis of human HNSCC cells

**Figure 5:**
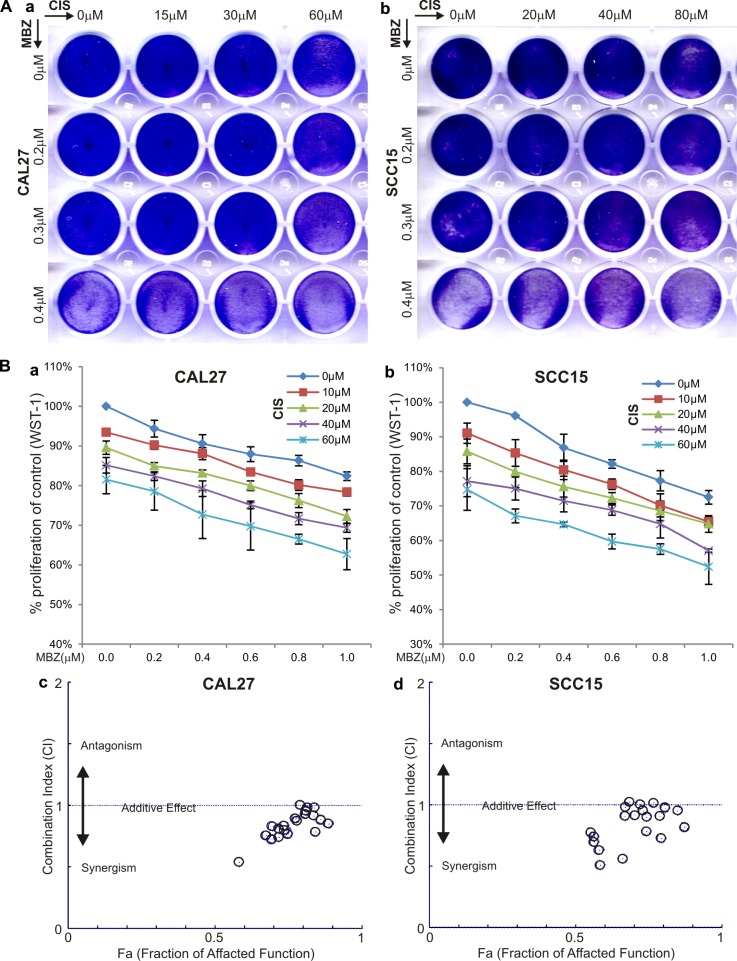
MBZ synergizes with cisplatin (CIS) in suppressing cell proliferation (**A**) Crystal violet staining assay. Subconfluent CAL27 (a) and SCC15 (b) cells were treated with the indicated concentrations of MBZ and CIS. At 72 h after treatment, the cells were fixed and stained with crystal violet. (**B**) WST-1 cell proliferation assay. Subconfluent CAL27 (a) and SCC15 (b) cells were treated with the indicated concentrations of MBZ and CIS for 48 h and incubated with premixed WST-1 reagent for 2 h before measuring absorbance. Quantitative evaluation of the synergy and combination index (CI) was conducted by using the Chou-Talalay method based on the WST-1 data obtained from CAL27 (c) and SCC15 (d) cells. CI < 1 indicates a synergism between MBZ and CIS.

MBZ was shown more potent than cisplatin in suppressing cell proliferation (Figure 1). Since cisplatin is currently used as a front line treatment of HNSCC cancers, we tested if MBZ and cisplatin would act synergistically to inhibit cell proliferation. Crystal violet staining assay indicated when CAL27 and SCC15 cells were treated varied concentrations of MBZ and/or cisplatin, there was a consistent trend showing more efficient inhibition of cell proliferation when both MBZ and cisplatin were presented (Figure 5A-ab). A more quantitative WST-1 assay revealed similar results in both CAL27 cells (Figure 5B-a) and SCC15 cells (Figure 5B-b). Furthermore, quantitative calculations of the combination index (CI) using the Chou-Talalay method [27] indicate that MBZ was shown to synergize with cisplatin (i.e., CI < 1) in both CAL27 cells (Figure 5B-c) and SCC15 cells (Figure 5B-d). Thus, these results suggest that MBZ may serve synergy partner with cisplatin in inhibiting cancer cell proliferation.

**Figure 6:**
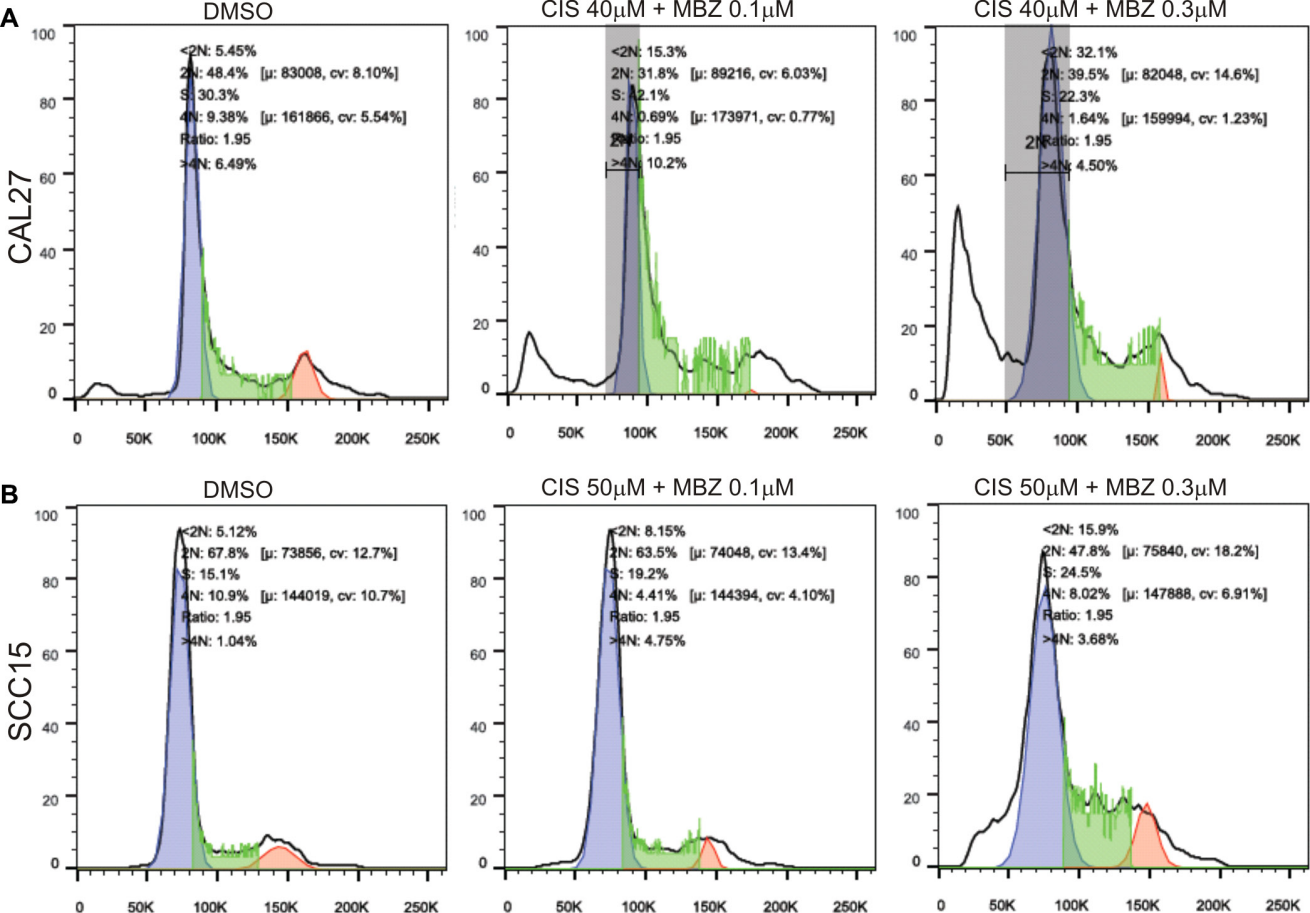
MBZ synergizes with cisplatin in suppressing cell cycle progression and inducing apoptosis Subconfluent CAL27 (**A**) and SCC15 (**B**) cells were treated with the indicated concentrations of MBZ and CIS for 48 h and stained with Hoechst 33258, followed by flow cytometry analysis. Each assay condition was done in triplicate. Representative results are shown.

The synergistic effect between MBZ and cisplatin was not only reflected in suppressing cell proliferation but also shown to effectively induce apoptosis. Hoechst 33258 staining-based flow cytometry analysis indicated a combination of lower concentration of 0.1 μM MBZ and 40 μM cisplatin achieved a similar apoptotic effect to that of a higher concentration of MBZ alone in CAL27 (Figure 6A). Similar synergistic pro-apoptotic effect was found in SCC15 cells both in a combination of lower concentration of 0.1 μM MBZ and 50 μM cisplatin and higher concentration of 0.3 μM MBZ and 50 μM cisplatin (Figure 6B). Thus, these results indicate that MBZ can synergize with cisplatin in inhibiting proliferation and inducing apoptosis of human HNSCC cells.

### MBZ inhibits cell proliferation and induces keratinization of CAL27 cells *in vivo*

**Figure 7:**
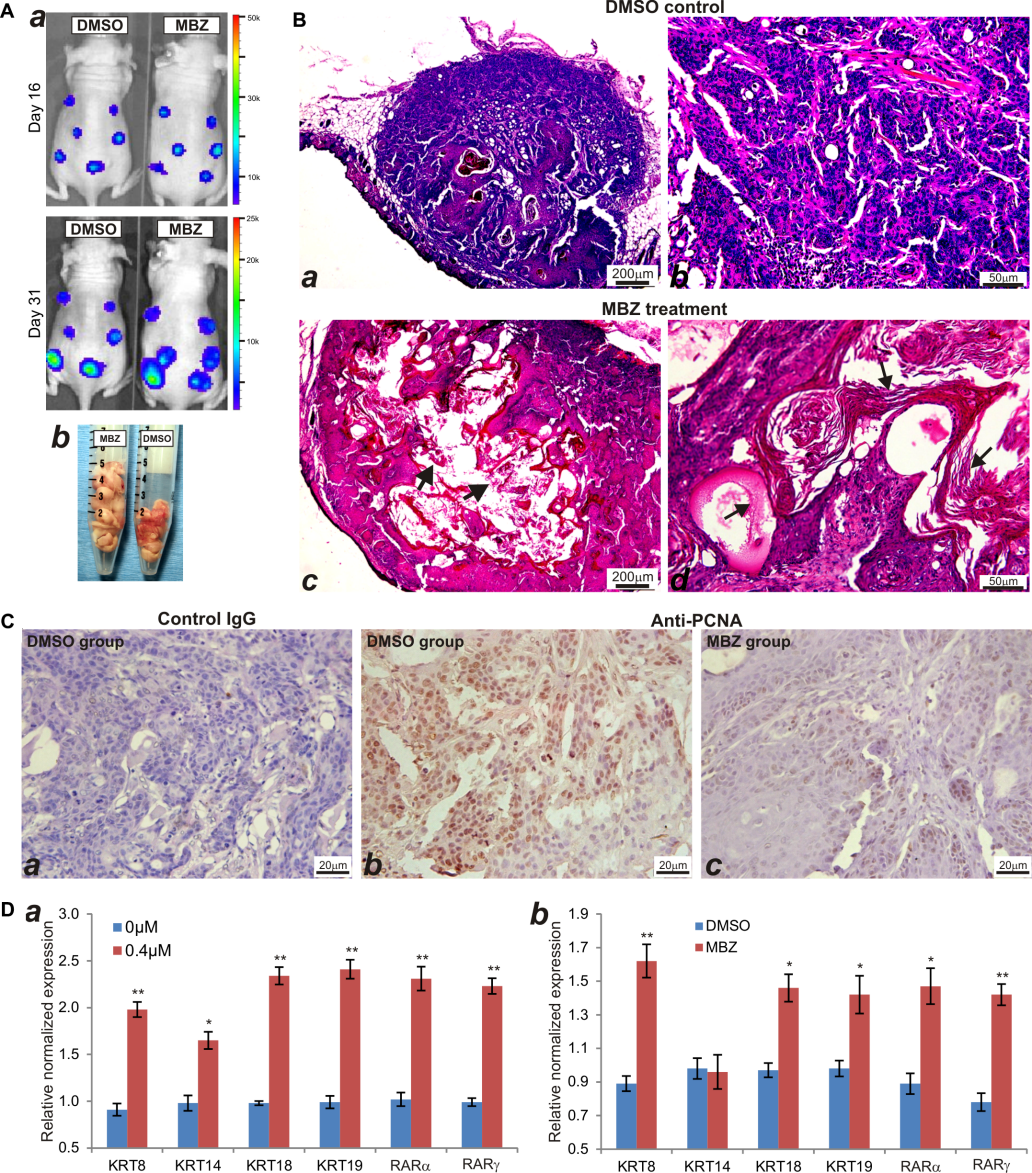
MBZ inhibits cell proliferation and induces keratinization of CAL27 cells in vivo (**A**) Xenogen bioluminescence imaging. The firefly luciferase-tagged CAL27 cells were injected subcutaneously into the flanks of athymic nude mice and Xenogen imaged at the indicated time points (**a**). At the endpoint, the mice were sacrificed and tumor masses were collected (**b**). (**B**) H & E staining of the retrieved tumor samples from the control group (a and b) and MBZ treatment group (**c** and **d**). Both representative lower (**a** and **c**) and higher (**b** and **d**) magnifications are shown. Typically keratinized tissues are indicated by arrows. (**C**) Immunohistochemical (IHC) expression analysis of cell proliferation marker PCNA. The retrieved tumor samples were section and subjected to IHC staining with either a negative control IgG (**a**) or PCNA antibody (**b** and **c**). Representative results are shown. (**D**) MBZ induces the expression of keratinocyte differentiation markers in CAL27 cells. Total RNA was isolated form the MBZ-treated CAL27 cells (**a**) or CAL27-derived xenograft tumors (**b**), and subjected to quantitative TqPCR using primers specific for the indicated genes. GAPDH was used as a reference gene. *p < 0.05, **p < 0.001.

We next sought to test the *in vivo* anti-cancer activity of MBZ in a xenograft tumor model of human HNSCC line CAL27 cells. We chose CAL27 line because this line exhibited rather unexpected activation of several cancer-related pathways upon MBZ treatment (Figure 4). When the firefly-luciferase-tagged CAL27 cells were injected subcutaneously and treated with MBZ or vehicle control, whole body bioluminescence imaging did not show any significant difference in signal intensity at both day 16 and day 31 after treatment (Figure 7A-a). In fact, the total volume of the tumor masses retrieved from the MBZ treatment group was shown larger than that of the control group's (Figure 7A-b). However, histologic evaluation of the retrieved tumor samples indicated that the MBZ-treated tumor samples exhibited extensive keratinization and significantly less cellular, compared with that of the control group's (Figure 7B ab *vs*. cd). Immunohistochemical staining further confirmed that MBZ-treated tumor cells exhibited significantly decreased or diminished expression of cell proliferation marker PCNA, which was shown highly expressed in the control group (Figure 7C). The *in vivo* data strongly suggest that, even though the tumor sizes may not be significantly reduced, MBZ may effectively inhibit tumor cell proliferation and promote keratinization and terminal differentiation of CAL27 cancer cells.

To confirm that MBZ can promote terminal differentiation of CAL27 cancer cells, we examined the expression of keratinocyte differentiation markers in MBZ-treated cells and xenograft tumors. When CAL27 cells were treated with MBZ, we found the expression of the differentiation markers, such as keratin 8, keratin 14, keratin 18, keratin 19, RARα and RARγ, was significantly up-regulated, compared with that of the control's (Figure 7D-a). Furthermore, we analyzed the expression levels of these markers in the tumor samples retrieved from MBZ or DMSO-treated animals, and found that except keratin 14 all of the other markers expressed significantly higher in MBZ-treated samples (Figure 7D-b). Thus, these results demonstrate that MBZ exhibits a strong differentiation-promoting activity in certain types of HNSCC cells, such as CAL27. It would thus be interesting to investigate the mechanism underlying MBZ-induced differentiation-promoting anticancer activity.

## DISCUSSION

HNSCC cancers are often associated with locoregional recurrence and lymph node metastasis, leading to a dismal 50% 5-year survival rate for the patients with advanced disease [28]. The survival rate is further compromised by the development of resistance to the frontline chemotherapeutic drugs such as cisplatin. Thus, there is an urgent need to find novel and effective therapies to combat HNSCC. We demonstrate two commonly used HNSCC lines CAL27 and SCC15 are relatively insensitive to cisplatin, and MBZ exhibits more potent anti-proliferation activity than that of cisplatin's in HNSCC cell lines, suggesting MBZ may be explored as a novel and efficacious anticancer agent for HNSCC cancers.

As an anthelmintic drug, MBZ has a long safety track record for its clinical use in children [10]. Several studies indicate that MBZ and/or its derivative flubendazole exerted anticancer activities in adrenocortical carcinoma [9], glioblastoma multiform [8], medulloblastoma [10], melanoma [11], leukemia and myeloma [12], non-small cell lung cancer [13, 14], cholangiocarcinoma [15], gastric cancer [16], colon cancer [17], and breast cancer [18–20]. However, the antitumor effect of MBZ in HNSCC has yet to be investigated. Here, we demonstrate that MBZ effectively inhibits cell proliferation and cell cycle progression, suppresses cell migration and induces apoptosis in both HNSCC lines CAL27 and SCC15. Our findings are supported by recent studies about MBZ's anticancer activity in several types of cancers. It was shown that MBZ inhibited the growth of adrenocortical carcinoma cells, as well as the invasion and migration of cancer cells *in vitro*, and metastases formation *in vivo* [9]. MBZ showed survival benefit in two preclinical models of glioblastoma multiform [8]. It was postulated that the antitumor activity of MBZ in medulloblastoma may be at least partially attributable to the inhibition of tumor angiogenesis [10]. We found that MBZ can effectively induce apoptosis and cause cell arrested in sub-G0/G1 phase in a dose-dependent fashion. These findings are consistent with other reports in which MBZ was shown to induce mitotic arrest and apoptosis by depolymerizing tubulin in non-small cell lung cancer cells [9, 13].

Like other small molecule drugs, MBZ likely exerts its functions by modulating multiple signaling pathways and/or cellular processes. By using a panel of previously well-characterized cancer-associated pathway reporter analyses [25, 29–31], we found at least four pathways, e.g., ELK1/SRF, AP1, STAT1/2, MYC/MAX, are significantly affected. Interestingly, these four pathways are modulated by MBZ differently in the two HNSCC lines, activated in CAL27 cells but inhibited in SCC15 cells. While it's expected that MBZ would inhibit these proliferation-related signaling pathways in most cancer lines such as SCC15, it's rather surprising to find out these four pathway reporters are activated upon MBZ treatment in CAL27. One possibility is that MBZ may target certain cellular events downstream of these pathways so these reporters can be feedback up-regulated upon MBZ treatment. This phenomenon has been widely reported for many targeted cancer therapies, such as for the BRAF inhibitors that can induce high levels of BRAF expression in BRAFi treated melanoma cells [32–34]. Nonetheless, it's unclear why MBZ can exert opposite effects on several signaling pathways in CAL27 and SCC15 cell lines. According to the publically available database on commonly used cancer cell lines canSAR (http://cansar.icr.ac.uk) [35], rather distinctive and diverse genomic changes among CAL27 and SCC15 lines were found. For example, CAL27 line harbors more than 1,038 gene mutations, compared with 530 gene mutations for SCC15 cells, while most of them are nonsense or missense mutations (Supplementary Table 2). Similarly, the gene copy number variations are also significantly different, as CAL27 line has 184 genes with gain and 326 genes with loss whereas SCC15 line has 244 genes with gain and 12 genes with loss. Furthermore, these two lines exhibit quite different mutation profiles of the genes involved in cell cycle control, apoptosis and cell proliferation (Supplementary Table 2). Nonetheless, it remains to be fully investigated about the exact mechanisms underlying the opposite responses to MBZ in these two cell lines.

The CAL27 line was originally established in 1982 and is one of the most commonly used HNSCC cancer lines [36]. In this study, we find that CAL27 cells exhibit some interesting features in response to MBZ treatment. As mentioned above, the reporters for ELK1/SRF, AP1, STAT1/2, MYC/MAX were unexpectedly activated in CAL27 cells upon MBZ treatment, suggesting that CAL27 cells may harbor certain unique molecular and genetic features. When the CAL27-derived xenograft tumors were treated with MBZ, we found that the tumors not only did not shrink but also grew slightly larger, compared with that of the control group's. However, histologic evaluation revealed the MBZ-treated tumors were largely keratinized with low cellular components, suggesting that MBZ may promote CAL27 cells to undergo terminal differentiation. This possibility was further confirmed by the MBZ-induced increased expression of keratinocyte differentiation markers Keratin-8, Keratin-14, Keratin-18, Keratin-19, RARα, and RARγ [37–39]. It was reported that overexpression of RARα and RARγ enhanced retinoic acids’ ability to induce Keratin 19 expression [40], and there was an inverse relationship between RARβ expression and squamous differentiation or keratinization [41, 42]. Thus, to the best of our knowledge our results are the time to demonstrate that MBZ can promote terminal differentiation of certain HNSCC cancer cells. Our results indicate that MBZ may exert more potent cytotoxicity and anticancer activity than that of the frontline chemotherapy drug cisplatin in HNSCC cells. Nonetheless, any effective clinical management of most cancers usually requires combination therapies. We demonstrate that MBZ exhibits a strong synergistic effect with cisplatin in inhibiting cell proliferation and inducing apoptosis in HNSCC cancer cells. Thus, it's conceivable that MBZ can be explored as a safe and efficacious agent to sensitize HNSCC cancers to cisplatin in order to maximize the anticancer efficacy and reduce the adverse effects of cisplatin, although thorough preclinical and clinical studies are required.

In summary, we demonstrate that MBZ potently inhibits cell proliferation and migration and induces apoptosis in the two commonly-used HNSCC cell lines CAL27 and SCC15. MBZ is shown to synergize anticancer activity with cisplatin. Mechanistically, MBZ can modulate several cancer-associated pathways, such as ELK1/SRF, AP1, STAT1/2, MYC/MAX, although the regulatory outcomes may be context-dependent. Furthermore, MBZ promotes the terminal differentiation of CAL27 cells and keratinization of CAL27-derived xenograft tumors. Our findings are the first demonstration that MBZ may exert its anticancer activity by inhibiting cell proliferation while promoting differentiation of certain HNSCC cancer cells. Thus, MBZ can be repurposed as a safe and effective agent used in combination with other frontline chemotherapy drugs such as cisplatin in HNSCC treatment.

## MATERIALS AND METHODS

### Cell culture and chemicals

Human HNSCC lines CAL27 and SCC15 were purchased from ATCC (Manassas, VA). The cells were maintained in complete Dulbecco's Modified Eagle's Medium (DMEM) containing 10% fetal bovine serum (FBS, Invitrogen, Carlsbad, CA), 100 units of penicillin and 100 μg of streptomycin at 37°C in 5% CO_2_ as described [43–45], Unless indicated otherwise, all chemicals were purchased from Sigma-Aldrich (St. Louis, MO) or Thermo Fisher Scientific (Waltham, MA).

### Crystal violet cell viability assay

Crystal violet staining assay was conducted as described [46–51]. Briefly, subconfluent CAL27 and SCC15 cells were treated with varied concentrations of cisplatin, mebendazole, or DMSO vehicle control. At 72 h after treatment, cells were gently washed with PBS and stained with 0.5% crystal violet/formalin solution at room temperature for 20–30 min. The stained cells were further washed with tape water and air-dried for taking macrographic images [52]. For quantitative measurement, the stained cells were dissolved in 10% acetic acid at room temperature for 20 min with shaking, followed by measuring absorbance at 570 nm using the microplate reader (BioTek EL800, Winooski, VT) [29, 53].

### WST-1 cell proliferation assay

Quantitative cell proliferation was assessed by using the Premixed WST-1 Reagent (Clontech, Mountain View, CA) as described [29, 53]. Briefly, subconfluent CAL27 and SCC15 cells seeded in 96-well plates were treated with mebendazole and/or cisplatin at the varied concentrations for 24 h or 48 h. The Premixed WST-1 Reagent was added to each well, followed by incubating at 37°C for 30 to 150 min and reading absorbance at 450 nm. Each assay condition was done in triplicate.

### Cell wounding/migration assay

Cell wounding and migration assay was performed as described [52, 54]. Briefly, freshly confluent HNSCC cells seeded in 35 mm cell culture dishes were wounded with sterile micro-pipette tips. At various time points, the wound healing status at the approximately same locations was recorded under a bright field microscope. Each assay condition was done in triplicate.

### Apoptosis analysis (Hoechst 33258 staining)

As previously described [29, 54], exponentially growing CAL27 and SCC15 cells were treated with varied concentrations of mebendazole or DMSO. At 24 h or 48 h post treatment, cells were collected, fixed and stained with the Magic Solution (10× stock: 0.5% NP-40, 3.4% formaldehyde, 10 μg/ml Hoechst 33258 in PBS). Apoptotic cells were examined and recorded under a fluorescence microscope. The results were repeated at least in three independent batches of experiments. The average numbers of apoptotic cells were calculated by counting apparent apoptotic cells in at least 10 random fields at 200× magnification for each assay condition.

### Cell cycle analysis

Cell cycle analysis was conducted as previously described [29]. The exponentially growing CAL27 and SCC15 cells were seeded in 60mm cell culture dishes and treated with varied concentrations of mebendazole or DMSO. At 24 h or 48 h post treatment, cells were collected and stained with the Magic Solution for 30 min. The stained cells were subjected to flow cytometry analysis using BD FACSCalibur-HTS. The acquired flow cytometry data were analyzed with FlowJo v10.0 software. Each assay condition was done in triplicate.

### Cell transfection and pathway reporter assay

Gaussia luciferase reporter assay was carried out as described [45, 55, 56]. The tested cancer-relate signaling pathway reporters, which were previously characterized, included HIF1A, ELK1/SRF, AP1, NFκB, STAT1/2, RBP-Jκ, MYC/MAX and TCF/LEF reporters [25, 29–31]. Experimentally, subconfluent CAL27 and SCC15 cells were seeded in 60 mm cell culture dishes and transfected with 5.0 μg/dish of each reporter plasmid using PEI (Polysciences, Warrington, PA). At 24 h post transfection, cells were replated in 24-well plates and treated with various concentrations of mebendazole or DMSO control. At 24, 48 or 72 h post treatment, culture media were taken and subjected to Gaussia luciferase assays using the BioLux Gaussia Luciferase Assay Kit (New England Biolabs). Each assay condition was done at least in triplicate.

### Total RNA isolation and touchdown-quantitative real-time PCR (TqPCR) analysis

Subconfluent cells were treated with varied concentrations of MBZ for 48 h and subjected to total RNA isolation samples using TRIZOL Reagents (Invitrogen). Total RNA was also isolated from the MBZ or DMSO-treated CAL27-derived xenograft tumor samples using TRIZOL Reagents. The isolated RNA was subjected to reverse transcription reactions with hexamer and M-MuLV reverse transcriptase (New England Biolabs, Ipswich, MA). The cDNA products were used as PCR templates. The qPCR primers were designed by using Primer3 program [57], and used to amplify the genes of interest (Supplementary Table 1). TqPCR was carried out by using SYBR Green-based qPCR analysis on a CFX-Connect unit (Bio-Rad Laboratories, Hercules, CA) as described [29]. The qPCR reactions were done in triplicate. *GAPDH* was used as a reference gene.

### Chou–Talalay drug CI analysis

The combination effects between mebendazole and cisplatin were calculated by using the Chou–Talalay method [27]. The dose-dependent curves of each drug alone and in combination were obtained through WST-1 proliferation assay. These data were then analyzed with the CompuSyn software (ComboSyn, Inc.). The calculated combination index (CI) offers the quantitative definition for additive effect (CI = 1), synergism (CI < 1), or antagonism (CI > 1) in drug combinations [27].

### Xenograft tumors of human CAL27 HNSCC cells

All animal experiments were carried out in accordance with the approved guidelines approved by the Institutional Animal Care and Use Committee. CAL27 cells were first stably transduced with firefly luciferase-expressing *piggBac* transposon vector pMPB4-Fluc [53, 58], resulting CAL27-FLuc. The high expression level of firefly luciferase was verified by luciferase assay using Promega's Firefly Luciferase Assay System. Eexponentially growing CAL27-Fluc cells were collected, resuspended at 10^7^ cells/ml and injected subcutaneously into the flanks of athymic nude mice (Harlan Laboratories, 5–6 week old, male, 3 × 10^6^ cells/injection, and 4~6 injections per mouse, 5 mice per group). The mice were randomly divided into two groups (*n* = 5 per group). At four days post injection, the animals were treated with mebendazole (7.5 mg/kg body weight) or vehicle control (DMSO) intraperitoneally once every two days. Tumor growth was monitored by whole body bioluminescence imaging using Xenogen IVIS 200 Imaging System at days 4, 16, and 31 after treatment. Mice were sacrificed at day 31 and subcutaneous tumor masses were retrieved for histologic evaluation and qPCR analysis.

### H & E staining and immunohistochemical (IHC) staining of xenograft tumor samples

All retrieved tumor masses were fixed in formalin, paraffin-embedded, and sectioned. The slides were deparaffinized, rehydrated and subjected to H & E staining [26, 59, 60]. IHC staining was performed as described [61–63]. Briefly, the sections were subjected to deparaffinization, followed by antigen retrieval and immunostaining with an anti-PCNA antibody (Santa Cruz Biotechnology). Control IgG and minus primary antibodies were used as negative controls.

### Statistical analysis

Quantitative data were expressed as mean ± standard deviation. Statistical significance was evaluated by means of SPSS (version 19.0, IBM Corp, Armonk, NY) [64] using one-way, two-way, or repeated ANOVA. S-N-K analysis between groups was also used when necessary. A *p <* 0.05 was considered statistically significant.

## SUPPLEMENTARY MATERIALS TABLES


