# Supplementary material for: Anthelmintic mebendazole enhances cisplatin's effect on suppressing cell proliferation and promotes differentiation of head and neck squamous cell carcinoma (HNSCC)
Source: Oncotarget. 2017 Jan 16;8(8):12968–82. doi: 10.18632/oncotarget.14673 (PMC5355070; doi:10.18632/oncotarget.14673)
Supplement: Supplementary file 1 [file oncotarget-08-12968-s001.pdf]

Anthelmintic mebendazole enhances cisplatin's effect on suppressing cell proliferation and promotes differentiation of head and neck squamous cell carcinoma (HNSCC)

Supplementary Materials

Supplementary Table 1: Primers used for qPCR analysis

Gene	Sequence		Accession Number
KRT14	Forward<br>Reverse	GCTGGTGGTGATGGGCTT<br>ACTTCCAGGTCGGCGTTG	NM_000526
KRT18	Forward<br>Reverse	AATGCCCGTCTTGCTGCT<br>CCAGCTGCAGTCGTGTGA	NM_000224
KRT19	Forward<br>Reverse	CGTATCCGTGTCCTCCGC<br>CAGGCGGTCGTTGAGGTT	NM_002276.4
KRT8	Forward<br>Reverse	GAGCAGATCAAGACCCTCAACA<br>AGCTCTCGAACATGTTGTCCAT	NM_001256282
RARα	Forward<br>Reverse	CAGCGACTCCTTGGACAGA<br>GGCAGAGGGGTGTCTTGAT	NM_000964
RARγ	Forward<br>Reverse	AACAAGGTGACCAGGAATCG<br>TGTCAGGTGACCCTTCTTCC	NM_000966

Supplementary Table 2: Partial list of genomic characteristics of CAL27 and SCC15 lines

	CAL27	SCC15
Origin	56yo male, tongue	55 yo male, tongue
Gene mutations (COSMIC)	1038	530
Copy number variation	184 genes with gain<br>326 genes with loss	244 genes with gain<br>12 genes with loss
ABL1	1379C > T<br>1322C > T	n.f.
APC	2311G > C	n.f.
BAD	79G > A	n.f.
CASP8	618_621delAATC	n.f.
CDH1	n.f.	1214A > G
CDKN1A	268G > T<br>166G > T	n.f.
CDKN2A	205G > T	144G > A
HIF1A	847G > C	n.f.
HNRNPL	1333C > A<br>1732C > A	n.f.
MAP1B	7375G > A	n.f.
MAP3K6	n.f.	2252A > G<br>2228A > G
NRAS	203G > C<br>274G > A	n.f.
NOTCH1	n.f.	6738C > G
PIK3R3	n.f.	1334A > G
PIK3CG	532C > T	n.f.
SMAD2	n.f.	827C > T<br>737C > T
SMAD4	733C > T	n.f.
SOX5	1640G > A	n.f.
SOX7	n.f.	908T > C
STAT4	n.f.	1558G > C
TGFBR1	134A > G	n.f.
TP53	578A > T<br>182A > T<br>215C > G<br>299A > T	393 + 1G > A<br>672 + 1G > A<br>215C > G
TP73	1233C > G<br>1086C > G	n.f.

Note: n.f., not found.
Source: canSAR: https://cansar.icr.ac.uk/cansar/about-cansar/
